# Country-specific intervention strategies for top three TB burden countries using mathematical model

**DOI:** 10.1371/journal.pone.0230964

**Published:** 2020-04-09

**Authors:** Soyoung Kim, Aurelio A. de los Reyes V, Eunok Jung

**Affiliations:** 1 Department of Mathematics, Konkuk University, Seoul, Republic of Korea; 2 Institute of Mathematics, University of the Philippines, Diliman, Quezon City, Philippines; Xavier University, UNITED STATES

## Abstract

Tuberculosis (TB) is one of the top 10 causes of death globally and the leading cause of death by a single infectious pathogen. The World Health Organization (WHO) has declared the End TB Strategy, which targets a 90% reduction in the incidence rate by the year 2035 compared to the level in the year 2015. In this work, a TB model is considered to understand the transmission dynamics in the top three TB burden countries—India, China, and Indonesia. Country-specific epidemiological parameters were identified using data reported by the WHO. If India and Indonesia succeed in enhancing their treatment protocols and increase treatment and treatment success rate to that of China, the incidence rate could be reduced by 65.99% and 68.49%, respectively, by the end of 2035. Evidently, complementary interventions are essential to achieve the WHO target. Our analytical approach utilizes optimal control theory to obtain time-dependent nonpharmaceutical and latent case finding controls. The objective functional of the optimal control problem includes a payoff term reflecting the goal set by WHO. Appropriate combinations of control strategies are investigated. Based on the results, gradual enhancement and continuous implementation of intervention measures are recommended in each country.

## Introduction

Tuberculosis (TB), an infectious disease caused by the bacillus *Mycobacterium tuberculosis*, is one of the top 10 causes of death globally and the leading cause of death by a single infectious pathogen. According to the Global Tuberculosis Report 2019, it is estimated that in 2018, 10 million individuals developed TB and that approximately 1.23 million (including HIV-positive) deaths were TB-induced. The severity of national TB epidemics varies significantly among countries. In most high-income countries, fewer than 10 new cases per 100,000 population were reported, whereas this number was increased to 150–400 cases in most of the 30 high TB burden countries. The 30 countries with the highest TB burden, as listed by the World Health Organization (WHO), accounted for 87% of the cases worldwide. Two third of the world’s TB cases occurred in India (27%), China (9%), Indonesia (8%), the Philippines (6%), Pakistan (6%), Nigeria (4%), Bangladesh (4%) and South Africa (3%). Considerable progress in TB treatment and cure has resulted in a steady decrease in the number of incident cases and deaths in recent years [1]. Notwithstanding these developments, TB disease continues to be a major public health concern in many countries. The “end” of TB as an epidemic remains an aspiration and a distant reality. The United Nations (UN) held its first high-level meeting on TB in September 2018, emphasizing the need for urgent action to accelerate the progress toward eliminating the epidemic by 2030. All the member states of the WHO and UN have committed to end the global prevalence TB, initially through their unanimous endorsement of the WHO’s End TB Strategy in May 2014, and then, through their adoption of the UN Sustainable Development Goals (SDGs) in September 2015. The End TB Strategy set specific targets of 95% and 90% reduction in TB deaths and incidence rates, respectively, by 2035 compared with the levels in 2015 [2].

Mathematical modeling is effective for understanding the spread and control of infectious diseases [3]. In the assessment of the potential impacts of novel interventions, epidemiological models of disease transmission constitute an essential resource for informed policy decisions by public health sectors [4]. The first mathematical model describing the epidemiological trend of TB was developed by Waaler *et al*. [5]. It consisted of five discrete difference equations. The model parameters were estimated from observed data sets. The first continuous TB model composed of ordinary differential equations (ODE) was constructed by Revelle *et al*. [6]. A simple three-compartment TB model was constructed by Blower *et al*. [7]. They developed it further into a five-compartment model to incorporate TB endogenous reactivation and relapse. The basic reproduction number, epidemic doubling time, and threshold population size were derived. Model development progressed to include multi-strains, exogenous reinfection, and age-structure, as investigated by Castillo *et al*. [8–10]. The multi-strain model was developed by Cohen and Murray to investigate multidrug-resistant TB [11]. The effectiveness of chemoprophylaxis in preventing TB progression was studied by Bhunu *et al*. [12]. Liu and Zhang constructed a model to investigate the effects of vaccination and treatment on the spread of TB disease [13]. They calculated the basic reproductive number and analyzed the stability of equilibria.

To recommend or design TB epidemic control programs, our modeling approach utilizes the optimal control theory. It is a branch of mathematics developed to obtain optimal methods to control a dynamic system [14–16]. The optimal control theory applied to TB models attracts modelers’ attention because it provides valuable insights to public health agents in decision and policy making. A pioneering work of Jung *et al*. considered a two-strain TB model to reduce the number of infected and latent individuals with resistant TB [17]. Two control strategies were proposed: a *case finding control*, which reduces the number of latent individuals who develop the disease [17, 18]; and a *case holding control*, which decreases the incidence of acquired drug-resistant TB [17, 19]. Case finding and case holding strategies involving chemoprophylaxis, detection, and treatment controls are also proposed to minimize the number of individuals with active TB [20, 21]. In [22, 23], optimal strategies considered reinfection and post-exposure interventions to minimize the number of active TB infectious and persistent latent individuals. Whang *et al*. recommended *distancing control* as a supplement to case finding and case holding controls. It represents the effort for minimizing the number of susceptible individuals who become infected (e.g., educational campaigns or isolation of infectious individuals), to reduce the number of latent and active infectious individuals [24]. The impacts of these three control strategies in reducing the number of TB infected and infectious individuals were investigated further in [25]. In addition, a higher TB budget allocation in the Republic of Korea was proposed. The latter TB model was modified and adapted to describe TB transmission in the Philippines and identify control strategies for mitigating the disease [26]. Apart from distancing and case holding, *latent and active case finding* controls are considered. It was recommended that the Philippine government should intensify efforts for achieving active case finding control, to effectively and efficiently convince active TB patients to undergo appropriate diagnosis and treatment. Gao and Huang also applied the optimal control theory to a TB model and proposed control strategies that minimize the disease burden and intervention costs [27]. A concise review on the application of the optimal control theory to TB models is available in [28].

In this work, various TB control strategies, both constant and optimal interventions, are investigated. Among the top three TB burden countries under consideration, China exhibits the highest treatment success probability [29] and has implemented a considerable number of effective treatment measures. For India and Indonesia, constant case holding and active case finding control efforts are explored by decreasing the treatment failure probability and increasing the treatment rate, respectively, to China’s epidemiological parameter values. The numerical results revealed that even combined constant controls failed to achieve the 2035 target set by the WHO. This prompted the use of optimal control techniques. In the present framework, the objective functional is effectively selected to achieve a specific goal with minimal cost. In particular, a payoff term consisting of a 90% reduction in TB incidence rate by 2035 (compared to the 2015 level) is incorporated.

This paper is organized as follows: A mathematical model for TB transmission dynamics is presented in the following section. The basic reproductive number $R_{0}$ is then computed for each respective country. The TB intervention strategies are introduced in this section. The estimated parameters are presented in the Results and Discussion section. The population growth and TB incidence rates by 2035 are projected using the model. Constant and optimal TB control strategies are also discussed in this section. Various control measures including distancing (nonpharmaceutical), latent and active case finding, treatment, and combinations thereof are delineated. The final section summarizes the findings and presents an outlook for future research directions.

## Materials and methods

### Data on the three TB burden countries

The population and TB data are used to identify epidemiological parameters of the top three TB burden countries. Data is retrieved from the WHO’s global tuberculosis database. For each country, the estimated total population and TB incidence rate per 100,000 individuals during the years from 2000 to 2017 are retrieved from WHO TB burden estimates. The treatment failure rate and TB-induced death rate are calculated based on the report on treatment outcomes [29].

### Mathematical model of TB

**Fig 1:**
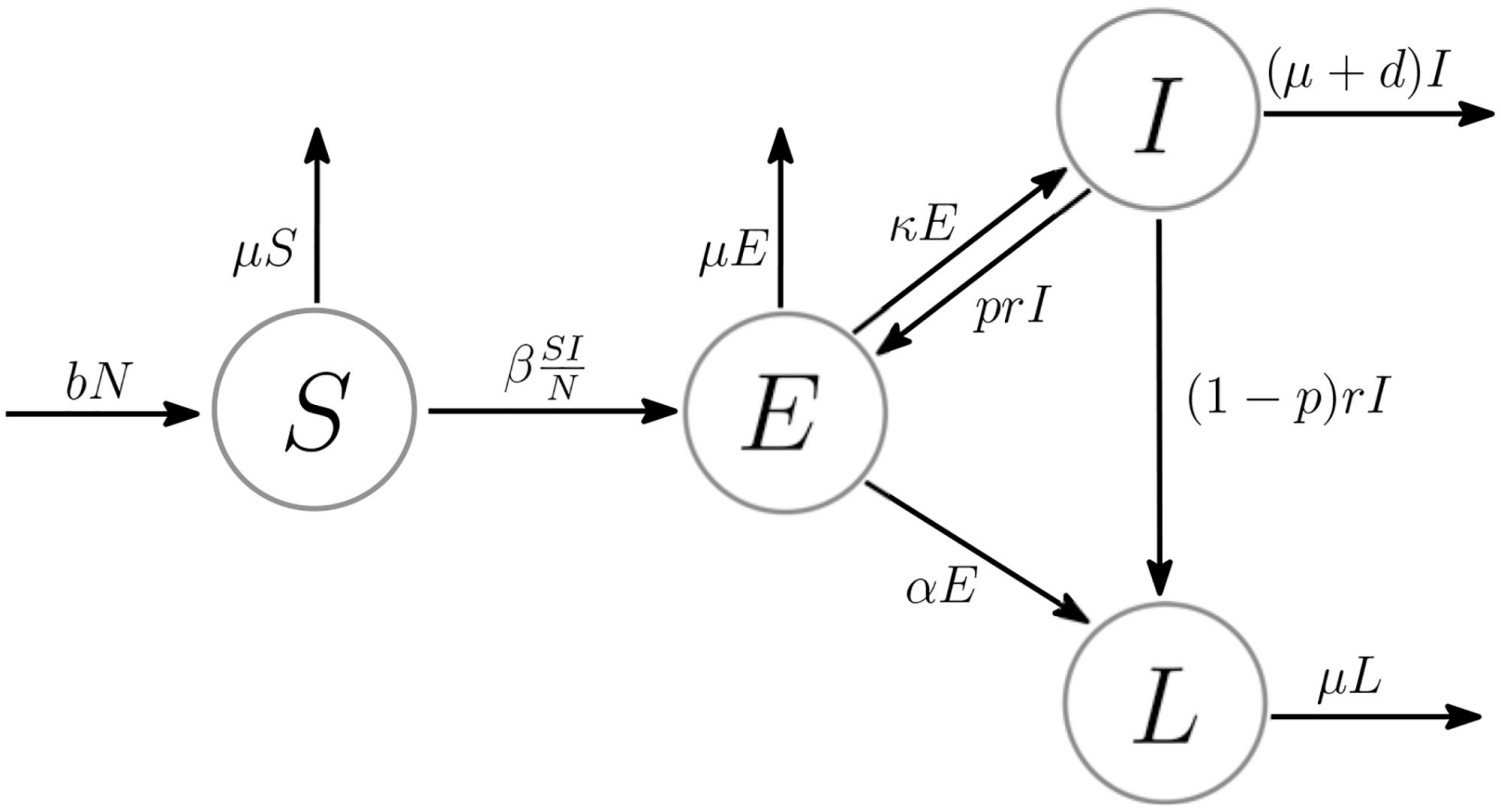
Flow diagram of TB dynamic model.

The TB model under consideration is adapted from our previous studies [24–26]. The total population (*N*) is divided into four epidemiological subgroups: susceptible (*S*), high-risk latent (*E*), active TB (*I*), and low-risk latent (*L*) individuals. Fig 1 displays the flow diagram of the TB transmission dynamics.

The susceptible individuals (*S*) moved to the high-risk latent group (*E*) through infection acquired from active TB patients (*I*). Not every infected individual is infectious. Those who are TB-infected but not infectious are called latent TB individuals, whereas infectious individuals are called active TB patients. There are no apparent symptoms during the latent period. A few individuals in the high-risk latent group progress to the active TB compartment. It is established that 5–15% of TB infected individuals exhibit a risk of falling ill with TB during their lifetime [30]. Note that *tubercle bacilli*, the TB-causing bacteria are not eliminated after the treatment. The bacilli remain in the body with a lower risk of progression to active TB. Individuals with TB bacilli who are low-risk latently infected are classified as low-risk latent individuals (*L*). However, the progression from low-risk latent to active TB is not considered in this work. The TB transmission dynamics is described by the following ODEs:

$$\begin{array}{c} \begin{array}{cc} \frac{dS}{dt} & =bN-\beta \frac{SI}{N}-\mu S\\ \frac{dE}{dt} & =\beta \frac{SI}{N}-\left(\alpha +\kappa +\mu \right)E+prI,\\ \frac{dI}{dt} & =\kappa E-(\mu +r+d)I,\\ \frac{dL}{dt} & =(1-p)rI+\alpha E-\mu L, \end{array} \end{array}$$ (1)

where *N* = *S* + *E* + *I* + *L*. The parameters *b* and *μ* represent the effective birth rate and natural death rate, respectively. The transmission rate is denoted by *β*. The parameter *κ* indicates the progression rate from the high-risk latent group to active TB, whereas *α* represents the progression rate from the high-risk to low-risk latent group. The treatment rate and TB-induced mortality rate are denoted by *r* and *d*, respectively. Active TB patients need to take regular medication for at least nine months to complete the TB treatment. However, anti-TB drugs induce mild to severe side effects and occasionally require a change in medication. Managing these side effects constitute a major factor in the adherence to TB treatment [31]. Failure to complete the treatment within the desired duration could result in undesirable TB drug-resistance. In this model, active TB patients who did not complete the TB treatment are denoted by *prI*. Here, *p* indicates the treatment failure rate. Therefore, 1 − *p* is the treatment success rate.

### TB intervention strategy

To eliminate the TB epidemic, various integrated intervention strategies have been implemented, such as health campaigns enhancing personal hygiene, detection of active TB patients, and monitoring of individuals to ensure completion of the TB treatment course. The TB intervention strategies can be classified into four categories: nonpharmaceutical control, latent case finding control, active case finding control, and case holding control. Nonpharmaceutical control encompasses all measures that do not employ medication. Educational health campaigns, quarantine, and isolation of active TB patients are included. In the present model, these efforts can be represented by decreasing the transmission rate *β*. Latent case finding control involves screening of high-risk latent individuals and their treatment to reduce the risk of progression. The treatment administration is called chemoprophylaxis. This control measure can be strengthened by increasing *α* in the model. The active case finding control entails effective detection, diagnosis, and cure of active TB patients. This control can be enhanced if *r* is increased. Case holding control is the effort for reducing the treatment failure rate *p*. Because of the side effect of the drugs and economic burden of the treatment, a few patients do not complete the treatment course. The directly observed treatment short (DOTS) course and financial support of TB treatment are included in case holding control.

The aim of this study is to recommend effective country-based intervention strategy to attain the percentage reduction in incidence rate level targeted by the WHO. The control needs to be prioritized to identify effective intervention strategies. It is inferred that controls aimed at smaller groups can be assessed as more efficient. For example, active case finding control focuses on individuals who exhibit TB-like symptoms, and case holding control monitors patients who are under treatment. The target population of these controls is specific and relatively small. Thus, control efficiency can be evaluated effectively. In contrast, latent TB case finding control requires screening of individuals not exhibiting TB symptoms. Essentially, this control considers the entire population to be screened for latent TB. Hence, it targets a large group, making the assessment more challenging.

In this work, constant intervention strategies, optimal time-dependent intervention strategies, and their combinations are investigated to achieve the End TB strategy by the WHO.

## Results and discussion

### Parameter estimation

**Fig 2:**
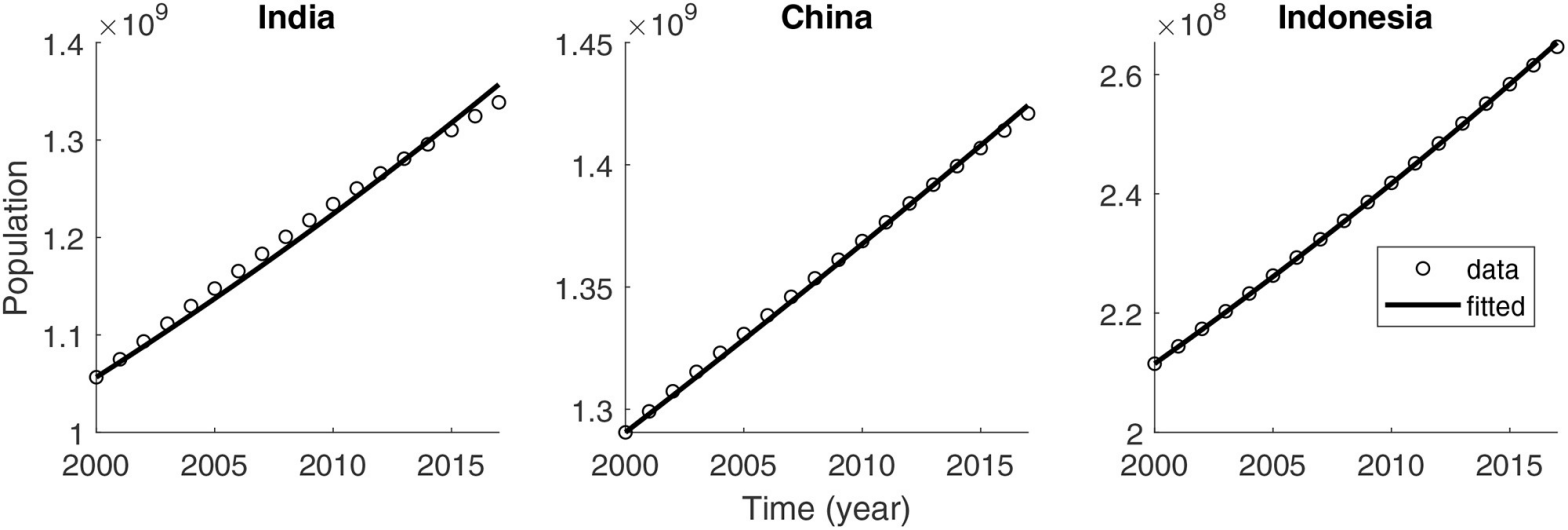
Total population data and the best fit of *N*(*t*) for India (left), China (middle), and Indonesia (right).

The epidemiological parameters are estimated using the data available for each country during the years 2000 to 2017 [29]. The effective birth and death rates are identified as follows. Adding the four ODEs in Eq (1) yields $\frac{dN}{dt}=bN-\mu N-dI.$ Because the number of TB-induced deaths (*dI*) is smaller than the other two terms (*bN* and *μN*), the equation can be approximated as $\frac{dN}{dt}\approx bN-\mu N$, with the solution *N*(*t*)≈*N*_0_ *e*^(*b*−*μ*)*t*^. The natural death rate *μ* is calculated using the life expectancy and the median age of the population. The effective birth rate *b* can be estimated by setting the population in 2000 as the initial population *N*_0_. The population net growth rate can then be computed as *b* − *μ*. Fig 2 displays the total population data (circles) and estimated curve (solid) for each country. The populations in three countries are growing: India (left), China (middle), and Indonesia (right). The net growth rate in India (0.0147) is the highest, followed by Indonesia (0.0133) and China (0.0058).

**Fig 3:**
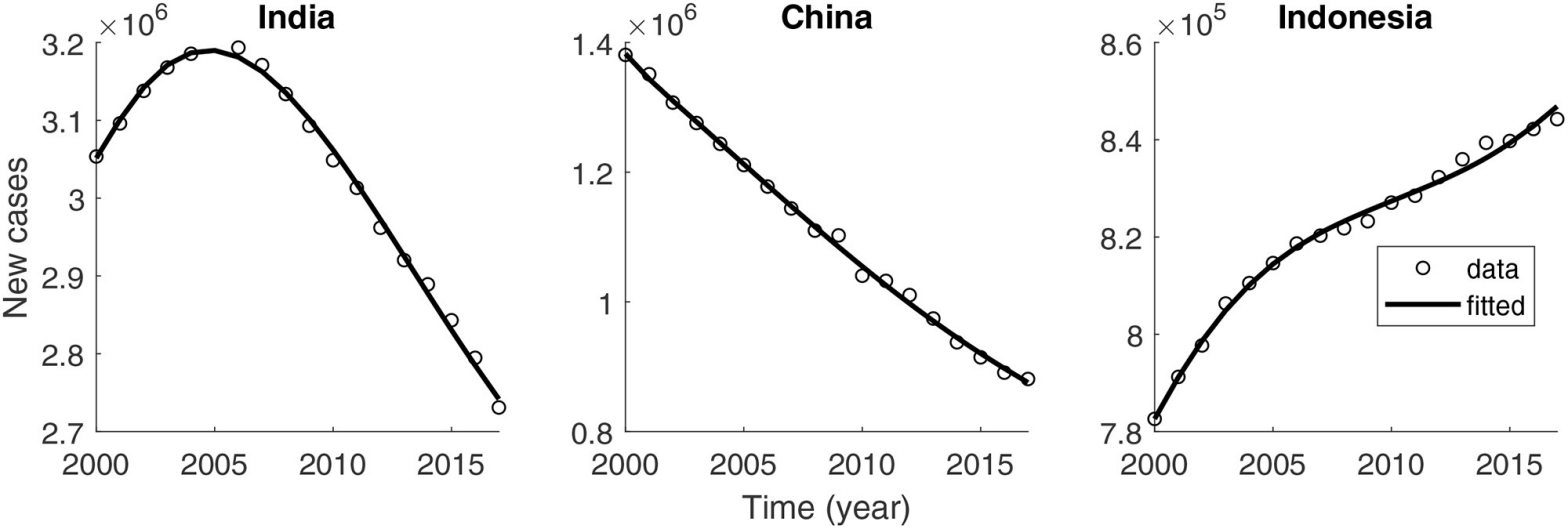
Reported numbers of incident cases (circle) and corresponding best fitted curves of *κE*(*t*) (solid) in top three TB burden countries.

The treatment failure rate *p* is calculated as the weighted average of the annual number of incident cases and treatment success rate reported by WHO. The TB-induced death rate is a weighted average value obtained using the annual number of incident cases and TB death rate. The transmission rate *β*, progression rate from high-risk to low-risk latent *α*, progression rate from high-risk latent to active TB *κ*, and treatment rate *r* are estimated by minimizing the error of the number of reported incident data to the model curve using the MATLAB routine fminsearch. Fig 3 depicts the number of incident cases (circles) and the estimated model curve (solid). The numbers of incident cases in India and China have declined in recent years, whereas it is still growing in Indonesia. The estimated epidemiological parameters and the initial proportion of high-risk and low-risk latent individuals are listed in Table 1.

**Table 1:** Epidemic parameters of the TB transmission model.

Parameter	Description	Value			References
		India	China	Indonesia	
*b*	effective birth rate	0.0394	0.0306	0.0373	data-fitted^†^
*μ*	natural death rate	0.0247	0.0248	0.0239	[32, 33]
*β*	transmission rate	10.7394	11.2590	10.1243	data-fitted^‡^
*α*	progression rate from *E* to *L*	0.4005	0.5938	0.3086	data-fitted^‡^
*κ*	progression rate from *E* to *I*	0.0500	0.0501	0.0500	data-fitted^‡^
*r*	treatment rate	0.4038	0.5422	0.2472	data-fitted^‡^
*d*	TB-induced mortality rate	0.1928	0.0609	0.1394	[29]
*p*	treatment failure probability	0.2250	0.0656	0.1267	[29]
$\frac{\left(E_{0}+L_{0}\right)}{N_{0}}$	initial proportion of high-risk and low-risk latent individuals	0.4000	0.3000	0.6600	[34] for India
					[35] for China and Indonesia

^†^ fitted from the total population data

^‡^ fitted from the incidence data

The infectious period is the average duration that individuals spend in the active TB state. It can be calculated by 1/(*r* + *μ* + *d*) in the mathematical model (1). The average infectious period is estimated as 1.6097 years in India, 1.5928 years in China, and 2.4357 years in Indonesia. Considering that the average treatment period for active TB is up to one year, the infectious period is relatively long. A few active TB individuals are unaware of their infection. They remain undiagnosed and spread TB to other susceptible individuals. This could imply that there is a time delay from being active TB to taking the treatment.

The basic reproductive number $R_{0}$ indicates the number of secondary cases from a primary case in the whole susceptible population. The value of $R_{0}$ indicates whether the disease will persist or not. If $R_{0}$ is lager than one, the number of cases increases and epidemic occurs. In contrast, the disease will die out if $R_{0}$ is less than one. From the mathematical model (1), $R_{0}$ is calculated using the next generation method [36, 37] as

$$\begin{array}{c} R_{0}=\frac{\beta \kappa }{(\alpha +\kappa +\mu )(\mu +r+d)-\kappa pr}. \end{array}$$

The estimated $R_{0}$ for Indonesia (3.2557) is the highest, followed by India (1.8463) and China (1.3485).

**Fig 4:**
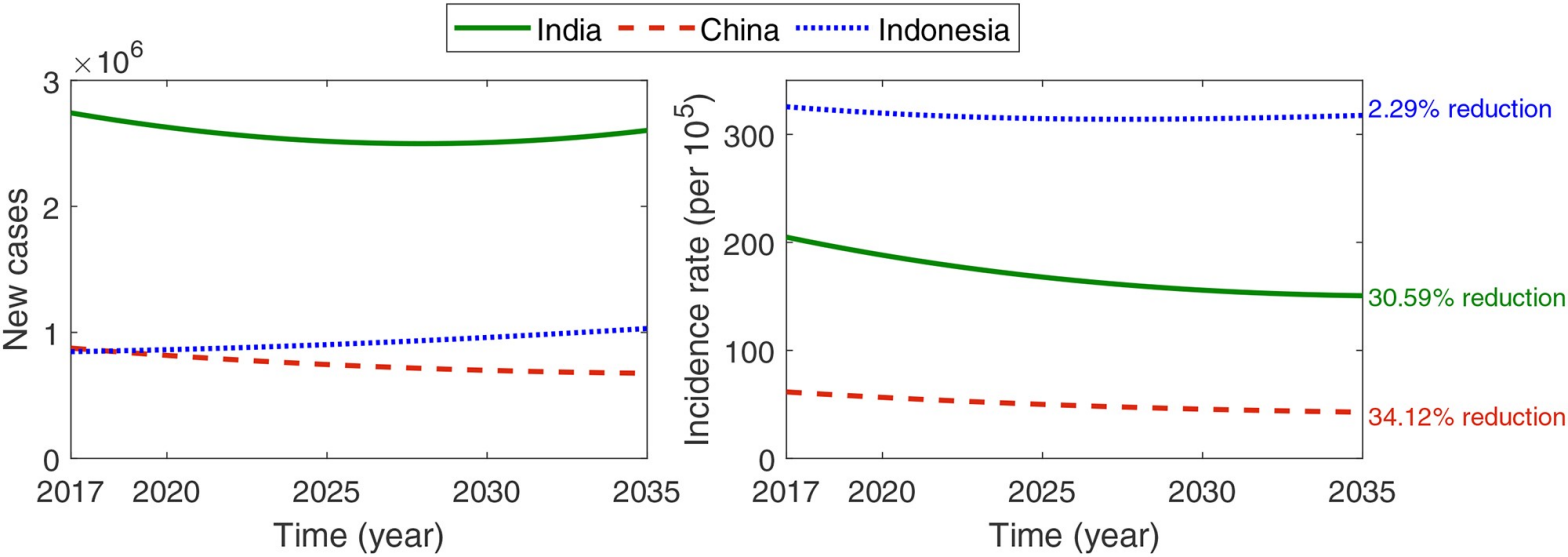
Expected number of incident cases (left) and incidence rate (right) from the TB model.

Fig 4 displays the expected numbers of new incident cases (left) and incidence rates (right) for the period till 2035, obtained from the TB model by using the identified epidemiological parameters listed in Table 1. The curves represent the projected trends assuming no significant improvement in the present TB intervention policies. The number of incident cases in India is the highest, whereas the incidence rate in Indonesia is the highest. Note that the number of incident cases in Indonesia will exceed that of China in a few years. Although the incidence rates of all three countries are decreasing, it is inadequate to satisfy the WHO’s End TB Strategy goal of 90% reduction by 2035 compared to the 2015 level. Hence, the present intervention strategies should be intensified, and/or additional controls should be implemented.

### TB constant control strategy

We first consider the case holding and active case finding controls with smaller target groups than those for the other controls. Among the three countries, China has superior TB treatment strategy, as indicated by the higher treatment rate *r* of approximately 0.5422 and treatment success rate (1 − *p*) of approximately 93%. We investigate the impact on the percentage reductions in the TB incidence rate in India and Indonesia if they follow China’s treatment intervention scheme. In particular, India’s and Indonesia’s epidemiological parameters *p* and *r* are replaced by China’s parameters. For clarity, the treatment failure and treatment rates in China are denoted by $\tilde{p}$ and $\tilde{r}$, respectively.

Three TB constant control strategies are considered for India and Indonesia: (C1) where only the case holding control strategy is intensified, i.e., *p* is decreased to $\tilde{p}$; (C2) where only the active case finding efforts are intensified, i.e., *r* is increased to $\tilde{r}$ and (C3), where both case holding and active case finding interventions are intensified, i.e., $\tilde{p}$ and $\tilde{r}$ are used. The expected percentage reductions in incidence rate compared to 2015 level are listed in Table 2. Note that the incidence rate per 100,000 is 217 in India, 65 in China, and 325 in Indonesia.

**Table 2:** Percentage reduction in the TB incidence rate.

Scenarios	Controls	India	Indonesia
C1	constant case holding ($\tilde{p}$)	33.44%	3.32%
C2	constant active case finding ($\tilde{r}$)	63.95%	67.65%
C3	constant case holding and active case finding ($\tilde{p}$, $\tilde{r}$)	65.99%	68.49%

**Fig 5:**
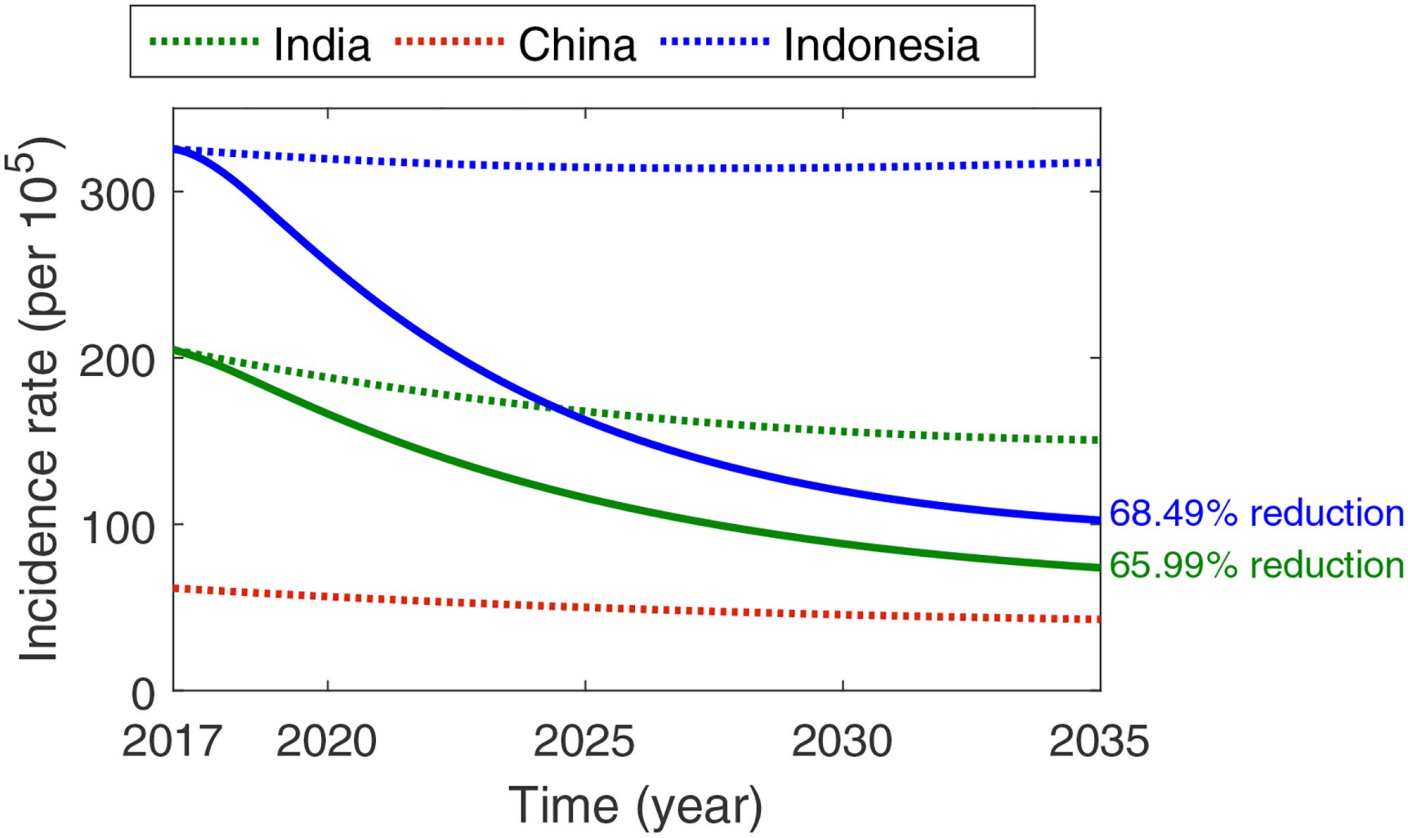
C3: Constant case holding and active case finding control strategies in India and Indonesia. The likely percentage reductions in the TB incidence rate are 65.99% and 68.49% in India and Indonesia, respectively. Note that no control strategy is applied in China.

Enhancing only case holding control in India and Indonesia is likely to yield percentage reductions in TB incidence rate of 33.44% and 3.32%, respectively. This strategy is subpar in mitigating TB in the two countries. It is noteworthy that an increase in the treatment success rate to approximately 93% yields an insignificant result for Indonesia owing to its relatively low treatment rate of approximately 0.2472. It can be inferred that because the proportion of active TB patients who are receiving the treatment is marginal, case holding control is less effective. When only the active case finding control is intensified, a considerable decrease in the TB incidence rate is likely: 63.95% and 67.65% reduction in India and Indonesia, respectively. Compared to constant case holding control C1, constant active case finding control C2 can reduce TB incidence rates further. Combining the two strategies is expected to yield incidence rate reductions of 65.99% in India and and 68.49% in Indonesia. The projected trends of incidence rates without control improvement (dotted) and with combined enhanced case holding and active case finding controls (solid) are depicted in Fig 5. Because no additional control strategy is applied in China, the percentage reduction is not shown.

Intensifying case holding and active case finding controls warrants reductions in the incidence rates in India and Indonesia, although insufficient to attain the End TB Strategy goal by 2035. Moreover, although China has a higher treatment rate and better treatment monitoring program, complementary interventions are required to achieve WHO’s target.

### TB optimal control strategy

Nonpharmaceutical and latent case finding controls are widely used TB intervention strategies. Optimal time-dependent controls can be obtained using the optimal control theory. The following is a system of a controlled TB model:

$$\begin{array}{c} \begin{array}{cc} \frac{dS}{dt} & =bN-(1-u_{1}\left(t\right))\beta \frac{SI}{N}-\mu S\\ \frac{dE}{dt} & =(1-u_{1}\left(t\right))\beta \frac{SI}{N}-((1+u_{2}\left(t\right))\alpha +\kappa +\mu )E+prI,\\ \frac{dI}{dt} & =\kappa E-(\mu +r+d)I,\\ \frac{dL}{dt} & =\left(1-p\right)rI+(1+u_{2}\left(t\right))\alpha E-\mu L, \end{array} \end{array}$$ (2)

where *N* = *S* + *E* + *I* + *L*. Nonpharmaceutical control, denoted by *u*_1_(*t*), represents the effort for reducing the transmission rate of TB. Meanwhile, the latent case finding control, *u*_2_(*t*), increases the progression rate from *E* to *L*. It can be achieved by screening and treating the high-risk latent individuals. This is called a latent TB treatment or chemoprophylaxis. The optimal control problem is formulated considering the End TB Strategy target of 90% reduction in TB incidence rate by 2035 compared to the 2015 baseline. Concurrently, the implementation costs should be minimized. Guided by these compelling aspects, our objective functional is defined as

$$J(u_{1}\left(t\right),u_{2}\left(t\right))=A{\left(E(T_{1})-C\right)}^{2}+\int_{T_{0}}^{T_{1}}(\frac{B_{1}}{2}u_{1}^{2}\left(t\right)+\frac{B_{2}}{2}u_{2}^{2}\left(t\right))\;dt,$$ (3)

where *T*_0_ and *T*_1_ are taken as the years 2017 and 2035, respectively. The parameter *C* represents the number of incidents at the final time *T*_1_ = 2035 satisfying the desired percentage reduction in the TB incidence rate. This value is dependent on the country’s information on the present and projected incidence cases and total population, and can be computed from

$$\begin{array}{c} \frac{IR_{2015}-\kappa C/N^{*}\left(T_{1}\right)}{IR_{2015}}\times 100=90\left(\%\right). \end{array}$$

*IR*_2015_ indicates the incidence rate in 2015 in each country. For simplicity, N*(*T*_1_) can be approximated using the state equations without control (refer to Eq (1)). The parameters *A*, *B*_1_, and *B*_2_ are weight constants balancing the size and importance of terms in the objective functional (3). It depends on the incidence rate at *T*_0_ in each country. The parameter *A* is set as 10^8^/(*IR*_2015_)^2^ and *B*_1_ and *B*_2_ are equal to one.

The optimal controls $u_{1}^{*}\left(t\right)$ and $u_{2}^{*}\left(t\right)$ need to satisfy

$$\begin{array}{c} J(u_{1}^{*}\left(t\right),u_{2}^{*}\left(t\right))=\min_{\Omega }J(u_{1}\left(t\right),u_{2}\left(t\right)), \end{array}$$

where $\Omega =\left\{(u_{1}(t),u_{2}(t))|u_{\text{min}}\leq u_{i}(t)\leq u_{\text{max}},u_{i}\in L^{2}(2017,2035),\;i=1,2\right\}$. Parameters *u*_min_ and *u*_max_ are the upper and lower bounds of the controls and assumed to be 0.05 and 0.95, respectively. Pontryagin’s Maximum Principle [38] is used to solve the optimal control system. Characteristics of optimal control problems, including adjoint system, transversality condition, and optimality equations, are derived in the supplementary material.

The following intervention scenarios are considered.

- Scenario 1
  (*S*1.1) only nonpharmaceutical control (*u*_1_(*t*))
  (*S*1.2) nonpharmaceutical control with constant case holding and active case finding controls (*u*_1_(*t*), $\tilde{p}$, and $\tilde{r}$)
- Scenario 2
  (*S*2.1) only latent case finding control (*u*_2_(*t*))
  (*S*2.2) latent case finding control with constant case holding and active case finding controls (*u*_2_(*t*), $\tilde{p}$, and $\tilde{r}$)
- Scenario 3
  (*S*3.1) nonpharmaceutical and latent case finding controls (*u*_1_(*t*) and *u*_2_(*t*))
  (*S*3.2) nonpharmaceutical and latent case finding controls with constant case holding and active case finding controls (*u*_1_(*t*), *u*_2_(*t*), $\tilde{p}$, and $\tilde{r}$)

#### Scenario 1: Nonpharmaceutical control strategy

**Fig 6:**
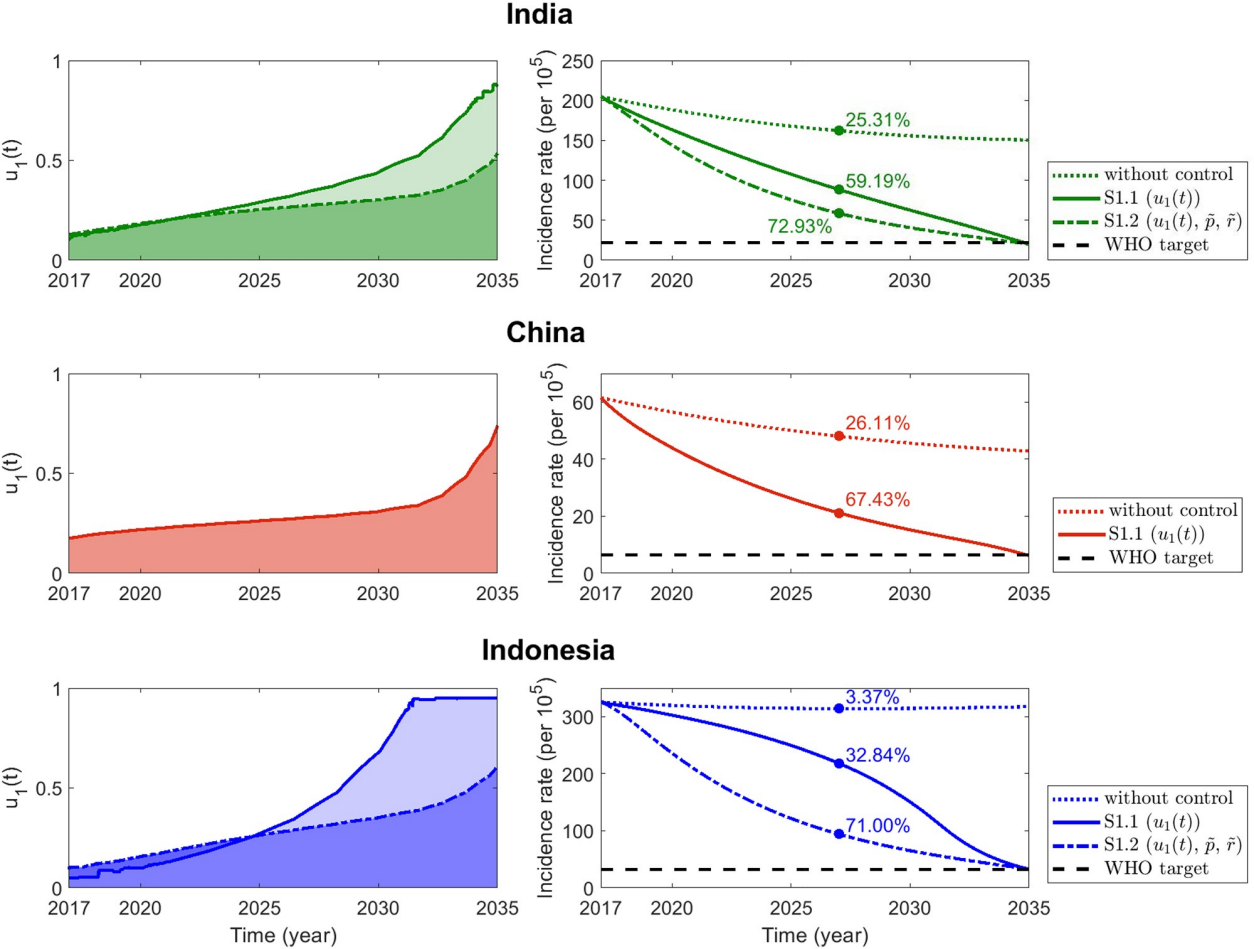
Scenario 1: Nonpharmaceutical control strategy. Optimal control strategies with constant case holding and active case finding controls (dash-dotted) or without the two constant controls (solid) are displayed as functions of time, in the frames on the left. The frames on the right present the corresponding incidence rates and the WHO targets in 2035 (black dashed line).

The obtained optimal nonpharmaceutical control *u*_1_(*t*) and corresponding projected trends for incidence rate during the years 2017 to 2035 are displayed in Fig 6. Only optimal nonpharmaceutical strategy (solid) coupled with constant case holding and case holding controls (dash-dotted) are depicted in the panels on the left. The corresponding impacts of the controls on the incidence rate are illustrated in the panels on the right (with identical curve attributes), including without additional control measures (dotted) and the WHO target of 90% reduction by 2035 (black dashed line). The circles in the frames on the right mark the predicted percentage reduction in the incidence rate in 2025. This is for a comprehensive comparison with the End TB strategy milestone of 50% reduction by the year 2025.

The frames at the top depict tenable TB control measures in India when the nonpharmaceutical strategy is implemented. When constant active case finding and case holding controls are applied additionally (left panel, green dash-dotted), the nonpharmaceutical control effort is less than that without the two constant controls (left panel, green solid). For both the cases, nonpharmaceutical control needs to be enhanced gradually. Control implementation will continuously decrease the incidence rate, so that it will eventually attain the WHO’s target in 2035. This is illustrated in the panel on the right. As would be anticipated, the incidence rate decreases faster when nonpharmaceutical control is coupled with the two constant controls. In both strategies, the expected percentage reductions in 2025 are 59.19% and 72.93%, indicating that the WHO’s 2025 milestone would be attained.

Only nonpharmaceutical control is considered for China (middle frames) because the country’s epidemiological parameter indicates high treatment and treatment success rates. As shown, the nonpharmaceutical effort should be intensified continuously to achieve the WHO 2035 target. The optimal control strategy also depicts that the WHO milestone in 2025 will be achieved satisfactorily (a percentage reduction in incidence rate of 67.43%).

The frames at the bottom display the control implementation and respective incidence rate reduction in Indonesia. Analogous to the case in India, the nonpharmaceutical control is implemented to a significantly lesser extent when coupled with constant case holding and active case finding controls. The optimal controls signify an increasing implementation effort to decrease the incidence rate appreciably and attain the WHO target by 2035. However, it is remarkable that nonpharmaceutical control will yield a 32.84% reduction in the incidence rate, i.e., the 50% WHO milestone for 2025 will not be achieved. In this case, nonpharmaceutical measures should be enhanced dramatically. In addition, implementation efforts should be maximized toward the last quarter of 2017–2035, to achieve the WHO 2035 goal.

#### Scenario 2: Latent case finding control strategy

**Fig 7:**
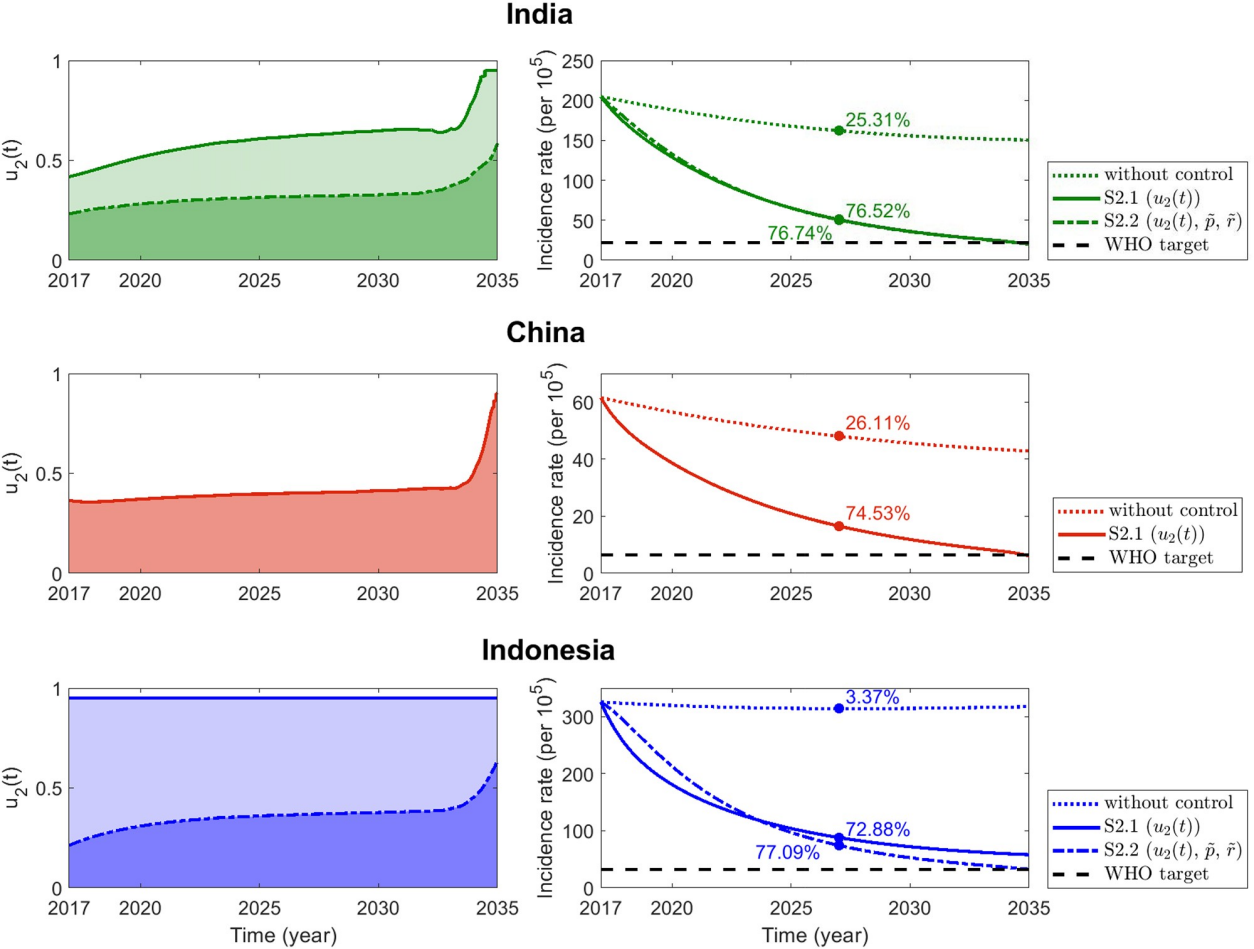
Scenario 2: Latent case finding control strategy. Optimal control strategies with constant case holding and active case finding controls (dash-dotted) or without the two constant controls (solid) are displayed as functions of time in the left frames. The right frames show the corresponding incidence rates and the WHO 2035 targets (black dashed line).

The optimal latent case finding control strategies for the top three TB burden countries are displayed in Fig 7. The optimal controls are depicted in the panels on the left, with the corresponding decreases in the incidence rates in the panels on the right.

The two frames at the top depict the latent control strategies for India and the expected reduction in TB incidence rate. The controls need to be strengthened regularly, with more effort and efficient implementation toward the end of the simulation time. In particular, without enhancement of active case finding and case holding controls (left panel, solid green), the latent case finding strategy should be implemented effectively with maximum effort as 2035 approaches. When two constant controls are additionally applied, less latent case finding measure is required (left panel, green dash-dotted). For both the cases, the projections for incidence rate reduction are similar, and the 90% decrease is achieved by 2035. The expected percentage reduction rates in 2025 are 76.52% and 76.74% in *S2.1* and *S2.2*, respectively. The two constant controls ($\tilde{p}$, and $\tilde{r}$), which target smaller number of individuals, complement the latent case finding control. Therefore, less effort will be required in the combined controls, compared to the use of only the latent case finding strategy.

In China, only the latent case finding control is considered for reducing the incidence rate (middle frames). The control measure is increased marginally throughout the period, but enhanced strongly toward the latter period. The percentage reduction in the incidence rate in 2025 is 74.53%. Compared to Scenario 1, latent case finding control is used more extensively than nonpharmaceutical control, yielding a faster incidence rate reduction.

The frames at the bottom display the optimal latent case finding control strategies and the projected incidence rate reduction in Indonesia. Observe that complete implementation and maximum usage of latent case finding control for the entire period (left panel, blue solid) are not adequate to reduce the incidence rate to 90% (right panel, blue solid). Additional constant case finding and case holding controls will scale down the effort required for implementing the latent case finding measure (left panel, blue dash-dotted). These combined strategies will enable the attainment of the WHO incidence rate reduction target in 2035 (right panel, blue dash-dotted). In 2025, the expected reduction rate without and with additional two constant controls are 72.88% and 77.09%, respectively. It appears that the final target in 2035 can be achieved without additional constant controls. However, the reduction speed becomes slower and eventually fails to attain the WHO goal. The complete implementation of latent case finding control represents an amplification by almost two times, of the progression rate from *E* to *L* indicated by *α*. The simulation result reveals that for Indonesia, increasing the progression rate to two times will not suffice for 90% reduction. Hence, using latent case finding as a single control should increase *α* to over two times, or other strategies need to be combined. Note that an incidence rate reduction is realized when only nonpharmaceutical control is implemented (see Scenario 1).

#### Scenario 3: Coupled control strategy

**Fig 8:**
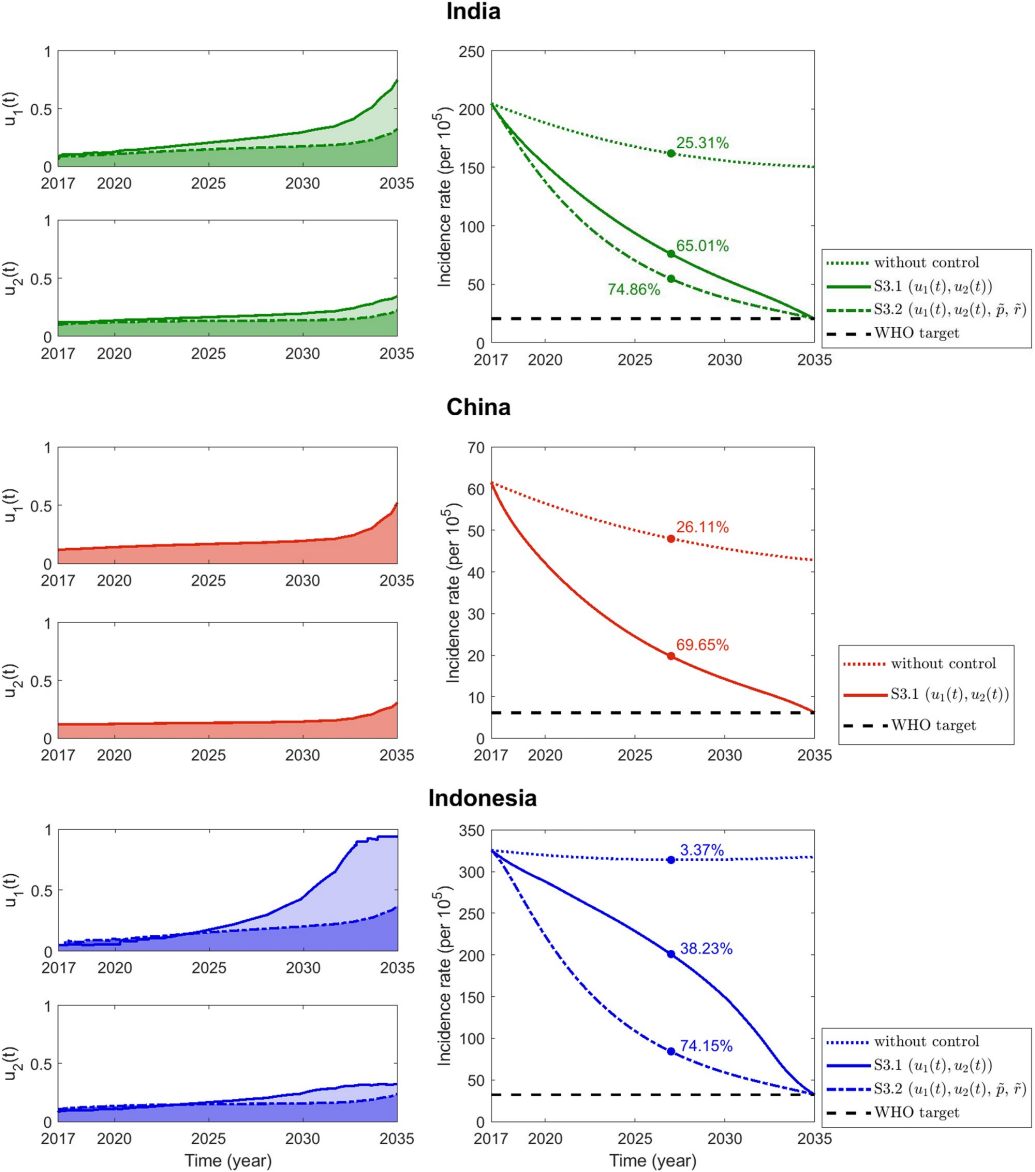
Scenario 3: Coupled control strategy. The left frames depict optimal control strategies with constant case holding and active case finding controls (dash-dotted) or without the two constant controls (solid), as functions of time. The right frames show the corresponding incidence rates and the WHO targets in 2035 (black dashed line).

Fig 8 displays the coupled optimal controls (left panels) and the corresponding incidence rate reductions (right panels) for the top three TB burden countries. The solid curves depict cases without additional constant controls, whereas the dash-dotted curves represent strategies coupled with constant active case finding and case holding controls.

The coupled control strategies for India are depicted in the frames at the top. In the absence of novel improvements in other control efforts, optimal coupled nonpharmaceutical and latent case finding strategies exhibit a gradual increase and more intensified efforts toward the end of the simulation duration (left panels, green solid). In contrast, the inclusion of constant active case holding and case finding controls exhibit a marginal increase in the optimal controls (left panels, green dash-dotted). Implementation of these coupled strategies can enable the attainment of the WHO’s 2025 milestone and its 2035 end TB goal, as depicted in the panel on the right. For China, a moderate enhancement of coupled optimal controls (middle, left panel) are required to achieve WHO’s TB milestone and target in 2025 and 2035, respectively (middle frame, right panels, solid red). In the case of Indonesia, coupled strategy requires more intensified efforts in nonpharmaceutical control with a significant increase in the middle of the simulation time and complete implementation toward the end (bottom frame, left panel, blue solid). Although this approach could enable the attainment of the 90% TB reduction target, the 2025 milestone is not attained with 38.23% expected incidence rate reduction (bottom frame, right panel, blue solid). If active case finding and case holding controls can be intensified and applied as constant controls, both nonpharmaceutical and latent case finding efforts will yield significant reduction (bottom frame, left panel, blue dash-dotted). Furthermore, both the 2025 milestone and 2035 target set by WHO will be realized (bottom frame, right panel, blue dash-dotted).

Evidently, coupled strategies with constant controls constitute the proposed intervention strategies for India and Indonesia. Efforts are appropriated among the different controls. Thereby, the burden of implementation is reduced, and significant incidence rate reductions with respect to the WHO 2025 milestone and 2035 target are achieved.

## Conclusion

Tuberculosis continues to be a major public health concern and poses a substantial economic burden in many countries. While there is a significant decrease in TB incident cases and deaths, TB epidemic elimination remains a far-fetched reality. The End TB Strategy set by WHO is aimed at achieving a 95% reduction in TB deaths and 90% decrease in incidence rates by 2035 compared with the 2015 baseline levels. Urgent actions are required to accelerate the progress toward the goal of ending TB. Consolidated efforts for prevention, improvement of coverage and quality of diagnosis, treatment, and care are significant measures toward achieving the WHO targets. These would entail unified activities from multi-sectors addressing social and economic determinants and consequences of TB, and those that enhance TB research and development, among others.

In this work, a dynamic TB model is considered to understand the transmission of the disease and determine potential interventions that could provide worthwhile insights for decision and policy making. The TB model under study is adapted to assess the top three TB burden countries: India, China, and Indonesia. Country-specific epidemiological parameters are identified using data reported by WHO and the estimated parameters are in the reasonable range of values. The identified model is utilized to capture the population growth trends and distinct TB incident cases among the countries. Furthermore, the model predicts the projected TB incidence rates in 2035.

The disparity in TB transmission among different countries as evidenced by the incidence data and fitted model demonstrates a demand for definitive control strategies suitable for each country. Because TB treatment success remains low (55% globally as reported by WHO [1]), practical attempts require the enhancement of case holding control (e.g., improvement of DOTS program and financial support for TB treatment) and intensification of active case finding control (e.g., higher coverage and higher quality of diagnosis, and screening the patients exhibiting TB like symptoms). In our dynamic model, strengthening these control measures entail decreasing treatment probability failure *p* and increasing the treatment rate *r*. Available data and model fitting signifies that China has superior case holding and active case finding control programs compared to India and Indonesia. It is observed that China has the highest treatment rate *r* and treatment success probability rate (1 − *p*). If India and Indonesia can increase their (1 − *p*) and *r* values to those of China, reduction in the incidence rates by 65.99% and 68.49% in India and Indonesia respectively, are likely by the year 2035.

The implementation of effective TB control strategies necessitates appropriate budget allocations. In low- and middle-income countries, financial constraints handicap policy implementation. The pressing task at hand is to reduce the number of incident cases with minimum intervention cost. Our modeling framework employs the optimal control theory to address this issue. The objective functional of the optimal control problem is selected so as to include a payoff term in conjunction with the controls. The payoff term reflects the WHO’s End TB Strategy target of 90% reduction in incidence rates by 2035 compared to the 2015 baseline level. The optimal control problem is formulated to determine effective TB intervention strategies that could achieve WHO’s goal by 2035 with the minimum economic burden. The control solution is increasing, unlike a typical control problem without payoff term in the objective functional. It indicates the need for gradual enhancement and continuous implementation of intervention measures, and increasing budget allocation toward the end of 2035. This is a more practical approach because full control implementation requires a challenging paradigm shift in monetary appropriation, particularly from poverty-stricken countries. For India and Indonesia, the application of constant controls for case holding and active case finding efforts by raising their (1 − *p*) and *r* values to those of China need to be complemented with nonpharmaceutical and latent case finding measures, to achieve the goal of WHO’s End TB Strategy. Note that even the present TB intervention policies in China, which has high treatment rates, require additional control efforts for the attainment of the WHO’s target by 2035. Optimal control techniques are applied to obtain nonpharmaceutical and latent case finding intervention profiles. Various scenarios are considered. As anticipated, a combination of the different controls will yield more desirable outcomes. The results reveal the model’s adaptability and its capability of designing intervention strategies based on a country’s epidemiological parameters. Based on the optimal control results, it can be inferred that a strategic pipeline is to start/continue with modest control interventions, monitor and assess the programs annually, continuously develop innovative diagnostics and treatment regiments, and increase sustain funding. This is in accordance with the WHO’s action plan to accelerate TB elimination [2].

The effectiveness of control measures are influenced by social, economic, cultural, geographical, and other factors. Furthermore, different countries have diverse priorities and budgetary limitations. Therefore, intervention strategies should be adapted based on a country’s specific needs. Further iterations of this modeling effort include a cost-effective analysis of the controls. It is effective to obtain better estimates on budget appropriation for mitigation programs of high TB burden countries. This could provide specific, efficient and practical intervention strategies. It has been reported that individuals living with HIV are 20–30 times more likely to develop active TB. Approximately 251,000 HIV-associated TB deaths and an estimate of 862,000 new TB cases among HIV-positive individuals have been identified [1]. Therefore, further model development should include the impact of TB–HIV coinfection in the transmission dynamics and corresponding mitigation strategies.

## Supporting information

S1 Appendix Characteristics of optimal control applied to TB model. Necessary conditions of optimal control.
(PDF)

## References

[1] World Health Organization. Global Tuberculosis Report 2019; 2018 Available from: https://www.who.int/tb/publications/global_report/en/.

[2] World Health Organization. Global Tuberculosis Report 2018; 2018 Available from: https://www.who.int/tb/publications/global_report/en/.

[3] Hethcote H. The Mathematics of Infectious Diseases. SIAM Review. 2000;42(4):599–653. 10.1137/S0036144500371907

[4] Garnett GP, Cousens S, Hallett TB, Steketee R, Walker N. Mathematical models in the evaluation of health programmes. The Lancet. 2011;378(9790):515–525. 10.1016/S0140-6736(10)61505-X 21481448

[5] Waaler H, Geser A, Andersen S. The use of mathematical models in the study of the epidemiology of tuberculosis. American Journal of Public Health and the Nations Health. 1962;52(6):1002–1013. 10.2105/AJPH.52.6.1002 PMC1523050 14004185

[6] Revelle CS, Lynn WR, Feldmann F. Mathematical Models for the Economic Allocation of Tuberculosis Control Activities in Developing Nations 1, 2. American Review of Respiratory Disease. 1967;96(5):893–909. 10.1164/arrd.1967.96.5.893 6059199

[7] Blower SM, Mclean AR, Porco TC, Small PM, Hopewell PC, Sanchez MA, et al The intrinsic transmission dynamics of tuberculosis epidemics. Nature medicine. 1995;1(8):815 10.1038/nm0895-815 7585186

[8] Castillo-Chavez C, Feng Z. To treat or not to treat: the case of tuberculosis. Journal of Mathematical Biology. 1997;35(6):629–656. 10.1007/s002850050069 9225454

[9] Castillo-Chavez C, Feng Z. Mathematical models for the disease dynamics of tuberculosis In: Arino O, Axelrod D, Kimmel M, editors. Advances in Mathematical Population Dynamics–Molecules Cells and Man. vol. 6 of Series in Mathematical Biology and Medicine. Singapore: World Scientific; 1998.

[10] Castillo-Chavez C, Song B. Dynamical models of tuberculosis and their applications. Mathematical biosciences and engineering. 2004;1(2):361–404. 10.3934/mbe.2004.1.361 20369977

[11] Cohen T, Murray M. Modeling epidemics of multidrug-resistant M. tuberculosis of heterogeneous fitness. Nature medicine. 2004;10(10):1117–1121. 10.1038/nm1110 15378056 PMC2652755

[12] Bhunu C, Garira W, Mukandavire Z, Zimba M. Tuberculosis transmission model with chemoprophylaxis and treatment. Bulletin of Mathematical Biology. 2008;70(4):1163–1191. 10.1007/s11538-008-9295-4 18231839

[13] Liu J, Zhang T. Global stability for a tuberculosis model. Mathematical and Computer Modelling. 2011;54(1-2):836–845. 10.1016/j.mcm.2011.03.033

[14] Pontryagin L, Boltyanskii V, Gramkrelidze R, Mischenko E. The Mathematical Theory of Optimal Processes. New York: Wiley Interscience; 1962.

[15] Fleming WH, Rishel RW. Deterministic and Stochastic Optimal Control. vol. 1 1st ed New York: Springer-Verlag; 1975.

[16] Cesari L. Optimization–Theory and Applications Problems with Ordinary Differential Equations. vol. 17 1st ed New York: Springer-Verlag; 1983.

[17] Jung E, Lenhart S, Feng Z. Optimal control of treatments in a two-strain tuberculosis model. Discrete and Continuous Dynamical Systems—Series B. 2002;2(4):473–482. 10.3934/dcdsb.2002.2.473

[18] Reichman LB, Hershfield ES. Tuberculosis: a comprehensive international approach. New York: Marcel Dekker, Inc.; 1993.

[19] Chaulet P. Treatment of tuberculosis: case holding until cure. World Health Organization, Geneva: WHO Tuberculosis Programme; 1983 Available from: https://apps.who.int/iris/handle/10665/58199.

[20] Bowong S. Optimal control of the transmission dynamics of tuberculosis. Nonlinear Dynamics. 2010;61(4):729–748. 10.1007/s11071-010-9683-9

[21] Bowong S, Alaoui AMA. Optimal intervention strategies for tuberculosis. Communications in Nonlinear Science and Numerical Simulation. 2013;18(6):1441–1453. 10.1016/j.cnsns.2012.08.001

[22] Silva CJ, Torres DFM. Optimal control strategies for tuberculosis treatment: A case study in Angola. Numerical Algebra, Control & Optimization. 2012;2(3):601–617. 10.3934/naco.2012.2.601

[23] Silva CJ, Torres DFM. Optimal control for a tuberculosis model with reinfection and post-exposure interventions. Mathematical Biosciences. 2013;244(2):154–164. 10.1016/j.mbs.2013.05.005 23707607

[24] Whang S, Choi S, Jung E. A dynamic model for tuberculosis transmission and optimal treatment strategies in South Korea. Journal of Theoretical Biology. 2011;279(1):120–131. 10.1016/j.jtbi.2011.03.009 21439972

[25] Choi S, Jung E. Optimal Tuberculosis Prevention and Control Strategy from a Mathematical Model Based on Real Data. Bulletin of Mathematical Biology. 2014;76(7):1566–1589. 10.1007/s11538-014-9962-6 24849770

[26] Kim S, de los Reyes AA, Jung E. Mathematical model and intervention strategies for mitigating tuberculosis in the Philippines. Journal of Theoretical Biology. 2018;443:100–112. 10.1016/j.jtbi.2018.01.026 29407656

[27] Gao Dp, Huang Nj. Optimal control analysis of a tuberculosis model. Applied Mathematical Modelling. 2018;58:47–64. 10.1016/j.apm.2017.12.027 PMC7117058 32287942

[28] Silva CJ, Torres DFM. Optimal Control of Tuberculosis: A Review In: Bourguignon JP, Jeltsch R, Pinto AA, Viana M, editors. Dynamics, Games and Science. Cham: Springer International Publishing; 2015 p. 701–722.

[29] World Health Organization. The WHO global TB data collection system; 2019 Available from: https://www.who.int/tb/country/data/download/en/.

[30] World Health Organization. Tuberculosis; 2018 Available from: https://www.who.int/news-room/fact-sheets/detail/tuberculosis/.

[31] Awofeso N. Anti-tuberculosis medication side-effects constitute major factor for poor adherence to tuberculosis treatment. Bulletin of the World Health Organization. 2008;86(3):B–D. 18368191 10.2471/BLT.07.043802 PMC2647396

[32] World Bank. Life expectancy at birth; 2019 Available from: https://data.worldbank.org/indicator/SP.DYN.LE00.IN.

[33] Nation Master. Countries Compared by People > Median age > Total. International Statistics at NationMaster.com; 2019. Available from: http://www.nationmaster.com/country-info/stats/People/Median-age/Total.

[34] Mahmood T. 40% of India’s population play host to the TB bacillus as a latent TB; 2016. Available from: https://www.oneindia.com/feature/40-percent-of-india-s-population-play-host-the-tb-bacillus-as-latent-tuberculosis-2049544.html.

[35] Houben RM, Dodd PJ. The global burden of latent tuberculosis infection: a re-estimation using mathematical modelling. PLoS medicine. 2016;13(10):e1002152 10.1371/journal.pmed.1002152 27780211 PMC5079585

[36] Diekmann O, Heesterbeek JAP, Metz JAJ. On the definition and the computation of the basic reproduction ratio R0 in models for infectious diseases in heterogeneous populations. Journal of Mathematical Biology. 1990;28(4):365–382. 10.1007/bf00178324 2117040

[37] van den Driessche P. Reproduction numbers of infectious disease models. Infectious Disease Modelling. 2017;2(3):288–303. 10.1016/j.idm.2017.06.002 29928743 PMC6002118

[38] Pontryagin LS. Mathematical theory of optimal processes. CRC Press; 1987.

