# Supplementary material for: Country-specific intervention strategies for top three TB burden countries using mathematical model
Source: PLoS One. 2020 Apr 9;15(4):e0230964. doi: 10.1371/journal.pone.0230964 (PMC7144981; doi:10.1371/journal.pone.0230964)
Supplement: S1 Appendix — Necessary conditions of optimal control. (PDF) [file pone.0230964.s001.pdf]

Supporting information

S1 Appendix. Characteristics of optimal control applied to TB model. Necessary conditions of optimal control.

The optimal control problem can be written as follows:

$$\min \left[A \left(\frac{kE(T_1)}{N^*(T_1)} - 0.1 \times IR_{2015} \right)^2 + \int_{T_0}^{T_1} \left[\frac{B_1}{2} u_1^2(t) + \frac{B_2}{2} u_2^2(t) \right] dt \right] \tag{1}$$

subject to

$$\begin{aligned}
\frac{dS}{dt} &= bN - (1 - u_1(t))\beta \frac{SI}{N} - \mu S \\
\frac{dE}{dt} &= (1 - u_1(t))\beta \frac{SI}{N} - ((1 + u_2(t))\alpha + \kappa + \mu)E + prI, \\
\frac{dI}{dt} &= \kappa E - (\mu + r + d)I, \\
\frac{dL}{dt} &= (1 - p)rI + (1 + u_2(t))\alpha E - \mu L, \\
&\text{where } N = S + E + I + L.
\end{aligned} \tag{2}$$

The goal is to reach the TB elimination goal of WHO with minimized control implementations (1). From Pontryagin's Maximum Principle [1], optimal controls should satisfy the necessary conditions. Pontryagin's Maximum Principle changes into problem that minimize pointwise a Hamiltonian H, with respect to the control.

Control strategies with $u_1(t)$ and $u_2(t)$ are considered to derive a necessary conditions for optimal control problem.

The Hamiltonian H is given as

$$\begin{aligned}
H =& \frac{B_1}{2} u_1^2(t) + \frac{B_2}{2} u_2^2(t) \\
&+ \lambda_1 \{ bN - (1 - u_1(t))\beta \frac{SI}{N} - \mu S \} \\
&+ \lambda_2 \{ (1 - u_1(t))\beta \frac{SI}{N} - ((1 + u_2(t))\alpha + \kappa + \mu)E + prI \} \\
&+ \lambda_3 \{ \kappa E - (\mu + r + d)I \} \\
&+ \lambda_4 \{ (1 - p)rI + (1 + u_2(t))\alpha E - \mu L \} \\
&+ \lambda_5 \{ bN - \mu N - dI \}.
\end{aligned}$$

Applying Pontryagin's Maximum Principle, we obtain the following theorem.

Theorem 1. *There exist optimal controls $u_1^*(t)$ and $u_2^*(t)$ minimizing the objective functional $\Omega = \{(u_1, u_2) \mid u_{\min} \le u_i(t) \le u_{\max}, u_i \in \mathcal{L}^2(2016, 2035),\ i = 1, 2\}$. Given these optimal solutions, there exist adjoint variables, $\lambda_1(t)$,..., $\lambda_5(t)$, which satisfy*

$$\begin{aligned}
\frac{d\lambda_1}{dt} =& \lambda_1 \big(\beta(1 - u_1)\frac{I}{N} + \mu \big) - \lambda_2 \beta (1 - u_1) \frac{I}{N}, \\
\frac{d\lambda_2}{dt} =& \lambda_2 \big(\kappa + \alpha(1 + u_2) + \mu \big) - \lambda_3 \kappa - \lambda_4 \alpha (1 + u_2) \\
\frac{d\lambda_3}{dt} =& \lambda_1 \beta (1 - u_1) \frac{S}{N} - \lambda_2 \big(pr + (1 - u_1)\beta \frac{S}{N} \big) + \lambda_3 \big(r + \mu + d \big) - \lambda_4 (1 - p) r + \lambda_5 d, \\
\frac{d\lambda_4}{dt} =& \lambda_4 \mu, \\
\frac{d\lambda_5}{dt} =& -\lambda_1 \big(b + (1 - u_1)\beta \frac{SI}{N^2} \big) + \lambda_2 (1 - u_1) \beta \frac{SI}{N^2} - \lambda_5 (b - \mu).
\end{aligned}$$

with transversality conditions $\lambda_1(T_1) = \lambda_3(T_1) = \lambda_4(T_1) = \lambda_5(T_1) = 0$*, and* $\lambda_2(T_1) = \frac{2Ak}{N(T_1)} \left(\frac{kE(T_1)}{N^*(T_1)} - 0.1 \times IR_{2015} \right)$.

Furthermore, the optimality equations are as follows

$$u_1^*(t) = min\left(u_{\max}, max\left(u_{\min}, (\lambda_2 - \lambda_1)\frac{\beta SI}{NB_1}\right)\right), \quad (3)$$
$$u_2^*(t) = min\left(u_{\max}, max\left(u_{\min}, (\lambda_2 - \lambda_4)\frac{\alpha E}{B_2}\right)\right).$$

Proof. The existence of optimal controls $u_1^*(t)$ and $u_2^*(t)$ such that $J(u_1^*(t), u_2^*(t)) = \min_\Omega(u_1(t), u_2(t))$ with state system is given by the convexity of objective functional integrand. By Pontryagin's Maximum Principle [1], the adjoint equations and transversality conditions are obtained. Differentiation of Hamiltonian H with respect to the state variables gives the following system:

$$\frac{d\lambda_1}{dt} = -\frac{\partial H}{\partial S}, \quad \frac{d\lambda_2}{dt} = -\frac{\partial H}{\partial E}, \quad \frac{d\lambda_3}{dt} = -\frac{\partial H}{\partial I}, \quad \frac{d\lambda_4}{dt} = -\frac{\partial H}{\partial L}, \quad \frac{d\lambda_5}{dt} = -\frac{\partial H}{\partial N},$$

with $\lambda_1(T_1) = \lambda_3(T_1) = \lambda_4(T_1) = \lambda_5(T_1) = 0$, and $\lambda_2(T_1) = \frac{2Ak}{N(T_1)} \left(\frac{kE(T_1)}{N^*(T_1)} - 0.1 \times IR_{2015} \right)$

Optimal controls $u_1^*(t)$ and $u_2^*(t)$ are derived by the following optimality conditions

$$\frac{\partial H}{\partial u_1} = B_1 u_1 + (\lambda_1 - \lambda_2)\frac{\beta SI}{N} = 0,$$
$$\frac{\partial H}{\partial u_2} = B_2 u_2 + (\lambda_4 - \lambda_2)\alpha E = 0,$$

at $u_1^*(t)$ and $u_2^*(t)$ on the set Ω. On this set

$$u_1^*(t) = (\lambda_2 - \lambda_1)\frac{\beta SI}{NB_1},$$
$$u_2^*(t) = (\lambda_2 - \lambda_4)\frac{\alpha E}{B_2}.$$

Taking into account the bounds on controls, we obtain the characterization of $u_1^*(t)$ and $u_2^*(t)$ in (3). □

References

1. Pontryagin LS. Mathematical theory of optimal processes. CRC Press; 1987.
